# Lenzimycins A and B, Metabolites With Antibacterial Properties From *Brevibacillus* sp. Associated With the Dung Beetle *Onthophagus lenzii*

**DOI:** 10.3389/fmicb.2020.599911

**Published:** 2020-10-30

**Authors:** Joon Soo An, Seong-Heon Hong, Elisabeth Somers, Jayho Lee, Byung-Yong Kim, Donghee Woo, Suk Won Kim, Hee-Jeon Hong, Shin-Il Jo, Jongheon Shin, Ki-Bong Oh, Dong-Chan Oh

**Affiliations:** ^1^Natural Products Research Institute, College of Pharmacy, Seoul National University, Seoul, South Korea; ^2^Department of Biological and Medical Sciences, Faculty of Health and Life Sciences, Oxford Brookes University, Oxford, United Kingdom; ^3^Department of Agricultural Biotechnology, College of Agriculture and Life Sciences, Seoul National University, Seoul, South Korea; ^4^ChunLab, Inc., Seoul, South Korea; ^5^Animal Welfare Division, Seoul Zoo, Seoul Grand Park, Seoul, South Korea

**Keywords:** antibiotic, *Brevibacillus*, *Bacillus*, dung beetle, lenzimycin, natural product

## Abstract

Symbiotic microorganisms associated with insects can produce a wide array of metabolic products, which provide an opportunity for the discovery of useful natural products. Selective isolation of bacterial strains associated with the dung beetle, *Onthophagus lenzii*, identified two strains, of which the antibiotic-producing *Brevibacillus* sp. PTH23 inhibited the growth of *Bacillus* sp. CCARM 9248, which is most closely related to the well-known entomopathogen, *Bacillus thuringiensis*. A comprehensive chemical investigation based on antibiotic activity discovered two new antibiotics, named lenzimycins A and B (**1**-**2**), which inhibited growth of *Bacillus* sp. CCARM 9248. The ^1^H and ^13^C NMR, MS, MS/MS, and IR analyses elucidated the structures of **1** and **2**, which comprised a novel combination of fatty acid (12-methyltetradecanoic acid), glycerol, sulfate, and *N*-methyl ethanolamine. Furthermore, the acid hydrolysis of **1** revealed the absolute configuration of 12-methyltetradecanoic acid as 12*S* by comparing its optical rotation value with authentic (*R*)- and (*S*)-12-methyltetradecanoic acid. In addition to inhibition of *Bacillus* sp. CCARM 9248, lenzimycins A and B were found to inhibit the growth of some human pathogenic bacteria, including *Enterococcus faecium* and certain strains of *Enterococcus faecalis.* Furthermore, the present study elucidated that lenzimycins A and B activated a reporter system designed to detect the bacterial cell envelope stress, thereby indicating an activity against the integrity of the bacterial cell wall.

## Introduction

Insects harbor symbiotic or pathogenic microorganisms; therefore, they have gained increasing interest as a reservoir for biotechnologically and pharmaceutically important microbes (Bode, 2010). In the past 20 years, several studies on insect-microbe interactions have reported on the role of bacterial natural products as molecular mediators in symbiotic insect systems (Cantley and Clardy, 2015; Pishchany and Kolter, 2020). There is an accumulating body of research which reports the insect symbiosis as biotechnological resources. For instance, Oh et al. (2009) analyzed the mutualistic associations in fungus-growing ant, *Apterostigma dentigerum* at the molecular level and found that the symbiotic bacterium, *Pseudonocardia*, produces dentigerumycin, a cyclic depsipeptide with highly modified amino acids, to selectively inhibit the associated parasitic fungus (*Escovopsis* sp.). Similarly, chemical ecological investigation of attine ants, *Acromyrmex rugosus* and their symbionts revealed a combination of antifungal compounds that inhibited the specialized fungal pathogen, *Escovopsis*. These small bacterial molecules were also evaluated as inhibitors against the human protozoan parasite *Leishmania donovani* (Ortega et al., 2019). It has also been reported that the symbiotic *Streptomyces* bacteria, located in specialized antennal glands, excrete antibiotics to protect wasp larvae from pathogenic fungal infestation (Kaltenpoth et al., 2005; Kroiss et al., 2010). Furthermore, studies have shown that the stingless bees, *Melipona scutellaris*, engage in defensive symbiosis with actinobacteria that produce antibacterial small molecules including the lobophorin, anthracycline, and cyclic hexadepsipeptide families, helping to protect their colonies against the entomopathogenic *Paenibacillus larvae* (Rodríguez-Hernández et al., 2019; Menegatti et al., 2020).

Dung beetles (Coleoptera: Scarabaeidae) are interesting Coleopteran insects, which feed on herbivores’ feces and make brood balls with feces for larval development. Although the ecology has not been studied much in most of the dung beetle species, their life cycle is closely linked with feces. Therefore, they may possess symbiotic relationships with chemically prolific bacteria, some of which may aid in control of the microbes in their ecosystems. In our previous studies, we have isolated a new tricyclic macrocyclic lactam, tripartilactam from the *Streptomyces* sp. found in the brood ball of a dung beetle species, *Copris tripartitus* (Park et al., 2012; Hwang et al., 2020). Furthermore, Kim et al. (2013) have isolated a *Streptomyces* strain producing tripartin, a novel dichlorinated indanone from the larva of *C. tripartitus*. Additionally, new alkenyl cinnamic acid-bearing cyclic peptides, coprisamides A and B (Um et al., 2015), and naphthoquinone-oxindole alkaloids, coprisidins A and B (Um et al., 2016), have also been discovered as natural products of bacterial isolates obtained from the gut of dung beetles.

**FIGURE 1:**
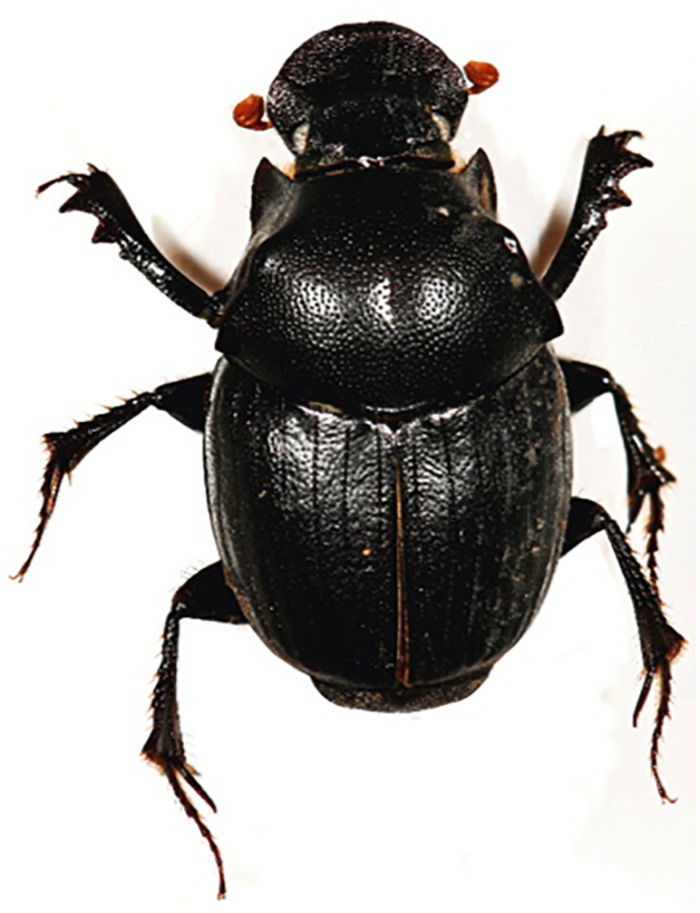
A specimen *Onthophagus lenzii*.

**FIGURE 2:**
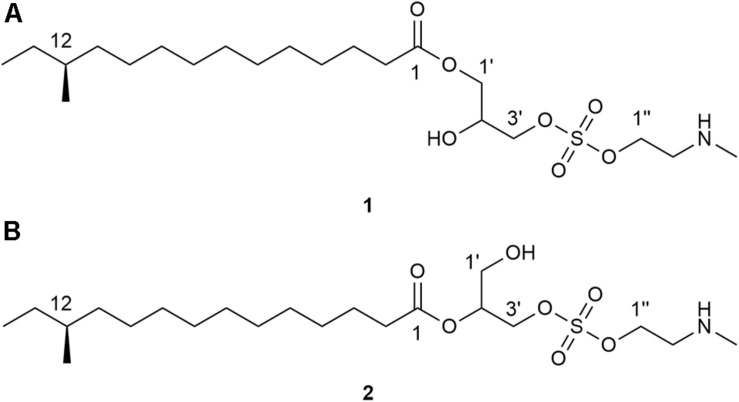
Structures of lenzimycins A and B (**1**-**2**).

Given the abundant species diversity of dung beetles and their ecological functions, it is expected to serve as a promising source in discovery of chemical compounds with potential pharmaceutical applications. In this study, we focused on a dung beetle species, *Onthophagus lenzii* (Yasuda, 1986), which is phylogenetically distant from *C. tripartitus*. The dung beetle *O. lenzii* (specimen shown in Figure 1) was collected from the city of Osan-si located on the outskirts of Seoul in South Korea. During the selective cultivation of bacteria using the excretion of the dung beetles, a *Brevibacillus* sp. PTH23 with an ability to inhibit a co-isolate, *Bacillus* sp. CCARM9247, which is a close relative of the well-known entomopathogen *Bacillus thuringiensis*, was isolated and further investigated to identify its antibiotic metabolites. Subsequent large-scale cultivation of PTH23 strain and chromatographic purification combined with a series of bioassay tests discovered two novel sulfated amino lipid antibiotics, lenzimycins A and B (Figure 2), which were also effective against some nosocomial human pathogenic bacteria such as *Staphylococcus aureus*, *Enterococcus faecium*, and *Enterococcus faecalis*. Furthermore, we also elucidated the structures and evaluated the biological functions of lenzimycins A and B (**1**-**2**).

## Materials and Methods

### General Experimental Procedures

Optical rotations were measured using a JASCO P-2000 polarimeter with a 1-cm cell. The ^1^H NMR spectra were measured with the Bruker Avance III HD 850 MHz spectrometer at the National Center for Inter-University Research Facilities (NCIRF) at Seoul National University; the ^13^C and 2D NMR spectra were recorded on the Bruker-800 MHz and JEOL JNM-ECA-600 MHz NMR spectrometers, respectively, at the College of Pharmacy, Seoul National University. Chemical shifts of all NMR spectra were referenced to the residual protonated solvent peaks for CDCl_3_ (δ_H_/δ_C_, 7.26/77.0). Low-resolution electrospray ionization (ESI) LC/MS data were acquired on an Agilent Technologies 6130 quadrupole mass spectrometer coupled with an Agilent Technologies 1200-series HPLC. High-resolution fast atom bombardment (HR-FAB) mass spectra were obtained using a JEOL JMS-600W high-resolution mass spectrometer at NCIRF. High-resolution electrospray ionization (ESI) LC/MS and ESI MS/MS data were obtained using an AB SCIEX Q-TOF 5600 high-resolution mass spectrometer at the National Instrumentation Center for Environmental Management (NICEM, Seoul, South Korea).

### Collection of *Onthophagus lenzii* (Harold)

A specimen of *O. lenzii* used in this study was collected from the entrance of the Doksanseong Fortress, Osan-si, Gyeonggi-do Province, South Korea, in March 2016. The adult dung beetle was secured inside a container using a tweezer and immediately transferred to the laboratory at Seoul National University. The collected specimen had a body length 6–12 mm and a glossy black body color, based on which it was identified as the dung beetle, *Onthophagus* (*Strandius*) *lenzii* Harold, 1874 (Coleoptera: Scarabaeidae). *O*. *lenzii*, widely distributed throughout Asia including Korea, Japan, and China, is the most dominant species among the genus *Onthophagus* (Coleoptera: Scarabaeidae: Scarabaeinae) in Korea (Kim, 2012). The prevailing appearance period of adults occurs annually from April to October, as they emerge from underground after an overwintering adult stage. These are the environmental cleanup insects, which eat and decompose the feces of cows, horses, and other large mammals (Yasuda, 1986). Moreover, the females of *O. lenzii* (Harold) make brood rooms and lay eggs under cow dung pats.

### Isolation of Bacterial Strains From the Dung Beetle

The regurgitated dark red excretion from the mouth of the dung beetle was scrapped by using a sterilized loop and a needle and diluted in 40 mL distilled water. The suspension was heated in a water bath at 55°C for 5 min. For bacterial isolation, 1 mL of the bacterial suspension was spread on each isolation agar medium (ISP1, ISP2, ISP4, CD, K, Chitin, and actinomycete isolation medium with cycloheximide) (Supplementary Table S1) and incubated at 30°C for 2 weeks. Based on their initial phenotypic observations, two types of colonies were selected and isolated from actinomycete isolation medium, and they were designated as PTH23 and CCARM924 (Supplementary Figure S15).

### Identification and Phylogenetic Analysis of Bacterial Isolates

The genomic DNAs of the isolated strains, PTH23 and CCARM9248, were extracted from the cell suspension cultures. The 16S rRNA gene was amplified by PCR with the bacterial universal forward primer 27F and reverse primer 1525R (Xu et al., 2003), and sequenced. Subsequently, similarity searches for the 16S rRNA gene sequences of the isolates were performed by the EzBioCloud server^1^ (Yoon et al., 2017) (GenBank accession number for PTH23: MT947196, and GenBank accession number for CCARM9248: MT947187), and the phylogenetic analysis was performed by the neighbor-joining method (Saitou and Nei, 1987) in the MEGA 7.0 program package (Kumar et al., 2016). Kimura’s two-parameter model was used to calculate the evolutionary distances (Kimura, 1980), and the bootstrap values were determined based on 1000 replications (Felsenstein, 1985).

### Cultivation and Extraction of PTH23

Based on the inhibitory zones, the strain PTH23 was presumed to have inhibitory effects on the growth of CCARM9248. Therefore, the PTH23 strain was further cultured on a large scale. First, the PTH23 strain was cultivated in 50 mL of ISP2 medium (4 g yeast extract, 10 g malt extract, and 4 g glucose in 1 L distilled water) in a 125-mL flask, and incubated for 2 days on a rotary shaker at 180 rpm at 30°C. Five milliliters of the broth culture was transferred to 200 mL of ISP2 medium in a 500-mL flask for initial scale up and incubated for 2 days on a rotary shaker at 160 rpm at 25°C. Then for the large-scale culture, 25 mL of the broth culture (obtained through initial scale up) was inoculated to 1 L of ISP2 medium in 2.8-L flasks (60 ea × 1 L, total volume 60 L) and incubated for 3 days on a rotator shaker at 160 rpm at 25°C. Further, the 60-L culture broth of the PTH23 strain was extracted with 90 L of ethyl acetate using a separation funnel. The residual water in the collected ethyl acetate layer was removed by adding anhydrous sodium sulfate. The extract was concentrated to dryness in a rotary evaporator *in vacuo* to obtain the final concentrated extract of 15 g.

### Purification of the PTH23 Extract

The extract of PTH23 was absorbed onto Celite, then loaded on a 2 g Sep-Pak C_18_ cartrige and fractionated with a step gradient solvent system (five fractions eluted with 100 mL each of MeOH-H_2_O from 20 to 100%). This process was repeated ten times to fractionate all the extract. After fractionation, lenzimycins A and B (**1**-**2**) were detected in the 80% MeOH-H_2_O fraction. The fraction eluted with 80% MeOH-H_2_O was filtered with a syringe filter (Advantec, 25HP045AN) and purified by semi-preparative reversed-phase HPLC column (YMC triart C_18_: 250 mm × 10 mm, 5 μm) with an isocratic solvent system [90% MeOH-H_2_O, refractive index (RI) detection, flow rate: 2 mL/min]. The antibiotics lenzimycins A (**1**) (3 mg) and B (6 mg) (**2**) were eluted at 18 min and 16 min, respectively.

#### Lenzimycin A (**1**)

Colorless powder; [α]${}_{\text{D}}^{25}$−5.2 (*c* 0.50, MeOH) IR (neat) *ν*_*max*_ 3274, 2924, 2853, 1735, 1633, 1464 cm^–1^; ^1^H and ^13^C NMR data, see Table 1; HRFABMS [M+H]^+^ *m*/*z* 454.2832 (calcd. for C_21_H_44_NO_7_S, 454.2838, error: −1.4 ppm).

**TABLE 1:** ^1^H and ^13^C NMR spectral data for lenzimycins A and B (**1**-**2**) in CDCl_3_.

**Position**	**1*^a^***				**2*^b^***			
	**δ_C_**	**Type**	**δ_H_,**	**mult (J in Hz)**	**δ_C_**	**Type**	**δ_H_,**	**mult (J in Hz)**
1	173.8	C			173.3	C		
2	34.1	CH_2_	2.30	t (7.7)	34.3	CH_2_	2.30	t (7.0)
3	24.9	CH_2_	1.60	m	24.9	CH_2_	1.60	m
4–10	30.0-27.1	CH_2_	1.31-1.23	m	30.0-27.1	CH_2_	1.31-1.23	m
11	36.6	CH_2_	1.27, 1.07	m	36.6	CH_2_	1.27, 1.07	m
12	34.4	CH	1.28	m	34.4	CH	1.28	m
12-Me	19.2	CH_3_	0.83	t (6.5)	19.2	CH_3_	0.84	t (6.5)
13	29.5	CH_2_	1.25, 1.12	m	29.5	CH_2_	1.25, 1.12	m
14	11.4	CH_3_	0.84	t (7.2)	11.4	CH_3_	0.85	t (7.2)
1′	64.5	CH_2_	4.11	m	59.8	CH_2_	3.73	d (5.3)
2′	69.1	CH	3.98	m	72.5	CH	4.97	m
3′	68.1	CH_2_	4.00, 3.90	m	63.4	CH_2_	4.08	m
1″″	61.6	CH_2_	4.19	m	61.2	CH_2_	4.20	m
2″	49.7	CH_2_	3.15	m	49.9	CH_2_	3.14	m
2″-NH			9.64	br s			9.73	br s
2″-N-Me	33.6	CH_3_	2.70	s	33.7	CH_3_	2.71	s

*^*a*1^H and ^13^C NMR were recorded at 800 MHz and 200 MHz, respectively.^*b*1^H and ^13^C NMR were recorded at 850 MHz and 212.5 MHz, respectively.*

#### Lenzimycin B (**2**)

Colorless powder; [α]${}_{\text{D}}^{25}$−1.0 (*c* 0.95, MeOH) IR (neat) *ν*_*max*_ 3398, 2925, 2853, 1733, 1643, 1466 cm^–1^; ^1^H and ^13^C NMR data, see Table 1; HRFABMS [M+H]^+^ *m*/*z* 454.2833 (calcd. for C_21_H_44_NO_7_S, 454.2838, error: −1.3 ppm).

### Acid Hydrolysis of Lenzimycin A (1)

To elucidate the absolute configurations of the fatty acid component, **1** (2.5 mg) was hydrolyzed in 0.5 mL of 6 N HCl and stirred at 120°C for 1 h. Then the reaction vial was cooled in an ice bath for 3 min. After the evaporation of the solvent *in vacuo*, the obtained dry material was dissolved in 0.5 mL of water and evaporated *in vacuo* for three times to remove residual HCl. The hydrolysate was purified by semi-preparative reversed-phase HPLC column (YMC triart C_18_: 250 mm × 10 mm, 5 μm) with an isocratic solvent system (90% MeOH-H_2_O, RI detection, flow rate: 2 mL/min). The desired product eluted at 31 min and confirmed as (*S*)-12-methyltetradecanoic acid (**3**, 0.5 mg) using ^1^H NMR and ESI-HRMS analyses.

#### (*S*)-12-Methyltetradecanoic Acid (**3**)

Colorless oil; [α]${}_{\text{D}}^{25}$ +5.6 (*c* 0.33, CHCl_3_) ESI-HRMS [M−H]^–^ at *m*/*z* 241.2167 (calcd for C_15_H_29_O_2_, 241.2162, error: 2.1 ppm) ^1^H NMR (600 MHz, CDCl_3_), δ_H_ 2.35 (2H, t, *J* = 7.4), 1.64 (2H, q, *J* = 7.4), 1.26 (17H, m), 1.12 (1H, m), 1.07 (1H, m), 0.86 (3H, t, *J* = 7.4), 0.84 (3H, d, *J* = 6.4).

### Minimum Inhibitory Concentration Assays Using Lenzimycins and Other Antibiotics

To investigate the antimicrobial activity of **1** and **2**, a series of minimal inhibitory concentration (MIC) tests was performed against various microbial strains, mostly the well-known nosocomial pathogens (Table 2). The MIC evaluation for the strains listed in Table 2A was carried out using a plate media dilution method (dilution range from 0.25 to 128 μg/mL) following the guidelines of Clinical Laboratory Standard Institute (Clinical and Laboratory Standards Institute [CLSI], 2018) and by using Mueller-Hinton I medium (BBL, Sparks, MD, United States). The MIC testing of the panel of four *E. faecalis* strains shown in Table 2B was performed in liquid culture using Brain Heart Infusion Broth (FLUKA). Several clinically important antibiotics, such as ampicillin, norfloxacin, vancomycin, teicoplanin, and bacitracin (Sigma-Aldrich) were used for comparative analysis of the antimicrobial activities of **1** and **2**.

**TABLE 2A:** Antibacterial activity of lenzimycins A and B (**1**-**2**) against a panel of bacterial species.

**Number**	**Strain**	**Accession number**	**Remarks**	**MIC (μg/mL)**			
				**1*^a^***	**2*^b^***	**AMP*^c^***	**NOR*^d^***
1	*Bacillus* sp.	CCARM 9248		64	8	16	0.12
2	*Bacillus cereus*	CCARM 0002		128	128	8	1
3	*Enterococcus faecalis*	CCARM 5171	susceptibl*^e^*	>128	>128	2	1
4	*Enterococcus faecalis*	CCARM 5025	VRE*^e^* (*vanB*)	>128	>128	2	2
5	*Enterococcus faecium*	CCARM 5024	VRE*^e^* (*vanA*)	0.5	1	32	4
6	*Staphylococcus aureus*	CCARM 0205	susceptible	32	64	2	1
7	*Staphylococcus aureus*	CCARM 3855	susceptible	>128	>128	0.25	1
8	*Staphylococcus aureus*	CCARM 3089	MDR*^f^*	>128	>128	64	128
9	*Escherichia coli*	CCARM 0230		>128	>128	2	0.03
10	*Escherichia coli*	CCARM 0237		>128	>128	8	0.5
11	*Escherichia coli*	CCARM 0235		>128	>128	4	0.06
12	*Pseudomonas aeruginosa*	CCARM 0219		>128	>128	>128	1
13	*Pseudomonas aeruginosa*	CCARM 0223		>128	>128	>128	0.5
14	*Pseudomonas aeruginosa*	CCARM 0225		>128	>128	0.5	0.25
15	*Salmonella Typhimurium*	CCARM 0240		>128	>128	1	0.06
16	*Enterobacter cloacae*	CCARM 0252		>128	>128	>128	0.06
17	*Enterobacter cloacae*	CCARM 0253		>128	>128	1	0.03
18	*Klebsiella oxytoca*	CCARM 0248		>128	>128	>128	0.03
19	*Klebsiella aerogenes*	CCARM 0249		>128	>128	2	0.06
20	*Escherichia coli*	ATCC 25922	QC*^g^* strain	>128	>128	4	0.06
21	*Enterococcus faecalis*	ATCC 29212	QC*^g^* strain	>128	>128	0.5	2 ∼ 8
22	*Enterococcus faecium*	ATCC 19434	QC*^g^* strain	16	8	0.5	–
23	*Pseudomonas aeruginosa*	ATCC 27853	QC*^g^* strain	>128	>128	>128	1 ∼ 4*^h^*
24	*Staphylococcus aureus*	ATCC 29213	QC*^g^* strain	>128	>128	0.13	0.5 ∼ 2*^h^*
25	*Salmonella enteric*	ATCC 14028	QC*^g^* strain	128	128	0.13	–
26	*Klebsiella pneumoniae*	ATCC 10031	QC*^g^* strain	>128	>128	32	–

**^*a*^* lenzimycin A, *^*b*^*lenzimycin B, *^*c*^*Ampicillin, *^*d*^*Norfloxacin, *^*e*^*Vancomycin-resistant *Enterococci*, *^*f*^*Multiple drug resistance, *^*g*^*Quality control, and *^*h*^*Acceptable range.*

**TABLE 2B:** Antibacterial activity of lenzimycins A and B (**1**-**2**) against additional clinical isolates of Enterococci.

**Strain**	**Remarks**	**MIC (μg/mL)**				
		**1*^a^***	**2*^b^***	**V^c^**	**T^d^**	**B^e^**
*Enterococcus faecalis* JH2-2	Parent strain, vancomycin sensitive	3.1	1.6	25	3.1	25
*Enterococcus faecalis* BM4316	JH2-2::Tn1546, inducible resistance, VanA-type VRE^f^	4.2	10.4	>100	>100	83
*Enterococcus faecalis* JH2-2::I	JH2-2::Tn1549, inducible resistance, VanB-type VRE^f^	6.3	6.3	>100	50	83
*Enterococcus faecalis* JH2-2::I C1	point mutation in *vanS*, constitutive resistance	4.2	6.3	100	83	83

**^*a*^* lenzimycin A, *^*b*^*lenzimycin B, *^*c*^*Vancomycin, *^*d*^*Teicoplanin, *^*e*^*Bacitracin, and ^*f*^Vancomycin-resistant *Enterococci*.*

### Bioassays for Activity Against the Bacterial Cell Wall

To elucidate the mechanism of action of **1** and **2**, the cell wall stress bioassay was performed. For the assay, approximately 10^7^ spores of the *Streptomyces coelicolor* reporter strain harboring plasmid pIJ6880 (Hong et al., 2002) were spread onto MMCGT agar medium supplemented with 80 μg/mL kanamycin; then, lenzimycins A and B dissolved in DMSO were applied to paper disks, and incubated for 2–4 days at 30°C. The antibiotics vancomycin (10 μg) and bacitracin (50 μg) were used as positive controls, whereas novobiocin (50 μg), that acts by targeting the DNA gyrase enzyme, was used as a negative control. DMSO (5 μL) was included as a solvent blank.

Additionally, we also performed the vancomycin synergy assay. The vancomycin-resistant strain *S. coelicolor* wild type M600 was spread onto MMCGT agar containing either 0 μg/mL or 10 μg/mL vancomycin, and the antibiotics were applied to paper disks. The results were scored after 2–4 days of incubation at 30°C. Any change in diameter of the dark zone of inhibition around the white paper disks was assessed by comparing the results on the plates containing and lacking vancomycin. Bacitracin (B, 50 μg) was used as a positive control, whereas teicoplanin (T, 5 μg) was used as a control for antagonism. Kanamycin (K, 5 μg), which acts through inhibition of protein synthesis by targeting the 30S subunit of rRNA, was used as a negative control.

## Results and Discussion

### Isolation of *Brevibacillus* sp. PTH23 From the Dung Beetle, *Onthophagus lenzii*

Two different types of isolates, PTH23, and CCARM9248, were identified from the excretion of the *O. lenzii*. As shown in Supplementary Figure S15, the colonies pertaining to PTH23 (middle of the clear circular zone of growth inhibition on the agar) inhibited the growth of CCARM9248, (growing around the zone of inhibition) on the isolation plate. Sequencing of the 16S rRNA genes and phylogenetic analysis revealed that the strains PTH23 and CCARM9248 shared high similarity with *Brevibacillus brevis* NBRC 15304^T^ (100%) and *B. thuringiensis* ATCC 10792^T^ (99.8%), respectively (Supplementary Figures S16, S17). We therefore designated them as *Brevibacillus* sp. PTH23 and *Bacillus* sp. CCARM9248. The zone of inhibition on the agar could be due to the production of antibiotic metabolites from *Brevibacillus* sp. PTH23 which inhibited the growth of the co-isolate, *Bacillus* sp. CCARM9248.

### Identification and Structure Elucidation of Key Metabolic Inhibitors of *Bacillus* sp. CCARM9248, Lenzimycins, From the *Brevibacillus* sp. PTH23 Extract

The *Brevibacillus* sp. PTH23 extract was fractionated by C_18_ reversed-phase column chromatography, and the fraction (80% MeOH-H_2_O), which displayed antibacterial activity, was analyzed by LC/MS. The chemical profile of the fraction revealed the presence of two metabolites, which had the molecular ions [M+H]^+^ commonly at *m*/*z* 454 in low-resolution ESI-MS analysis. Then, these compounds were further purified and designated as lenzimycins A and B (**1**-**2**) (Figure 2). Lenzimycin A (**1**) was obtained as a colorless oil. Its molecular formula C_21_H_43_NO_7_S, corresponding to one degree of unsaturation number, was deduced by the high resolution fast-atom bombardment mass spectroscopy (HR-FAB-MS) and NMR data (Table 1). The ^1^H NMR spectra of **1** (in CDCl_3_ at 800 MHz) identified one exchangeable proton (δ_H_ 9.64), seven protons attached to oxygenated carbons [δ_H_ 4.19 (2H overlapped), 4.11 (2H overlapped), 4.00, 3.98, and 3.90], five protons attached to a nitrogenous carbon [δ_H_ 3.15 (2H overlapped), 2.70 (3H)], twenty-three aliphatic protons (δ_H_ 2.30-1.12), and two aliphatic methyl groups (δ_H_ 0.84 and 0.83). The ^13^C and HSQC NMR data of **1** revealed the presence of one carbonyl carbon (δ_C_ 173.8), one oxymethine carbon (δ_C_/δ_H_ 69.1/3.98), three oxymethylene carbons (δ_C_/δ_H_ 68.1/4.00 and 3.90, 64.5/4.11, and 61.6/4.19), one methylene carbon bearing a nitrogen (δ_C_/δ_H_ 49.7/3.15), twelve *sp*^3^ methylene carbons (δ_C_/δ_H_ 36.6-24.9/2.30-1.23), one *N*-methyl group (δ_C_/δ_H_ 33.6/2.70), and two methyl carbons (δ_C_/δ_H_ 19.2/0.83 and 11.4/0.84) (Table 1).

**FIGURE 3:**
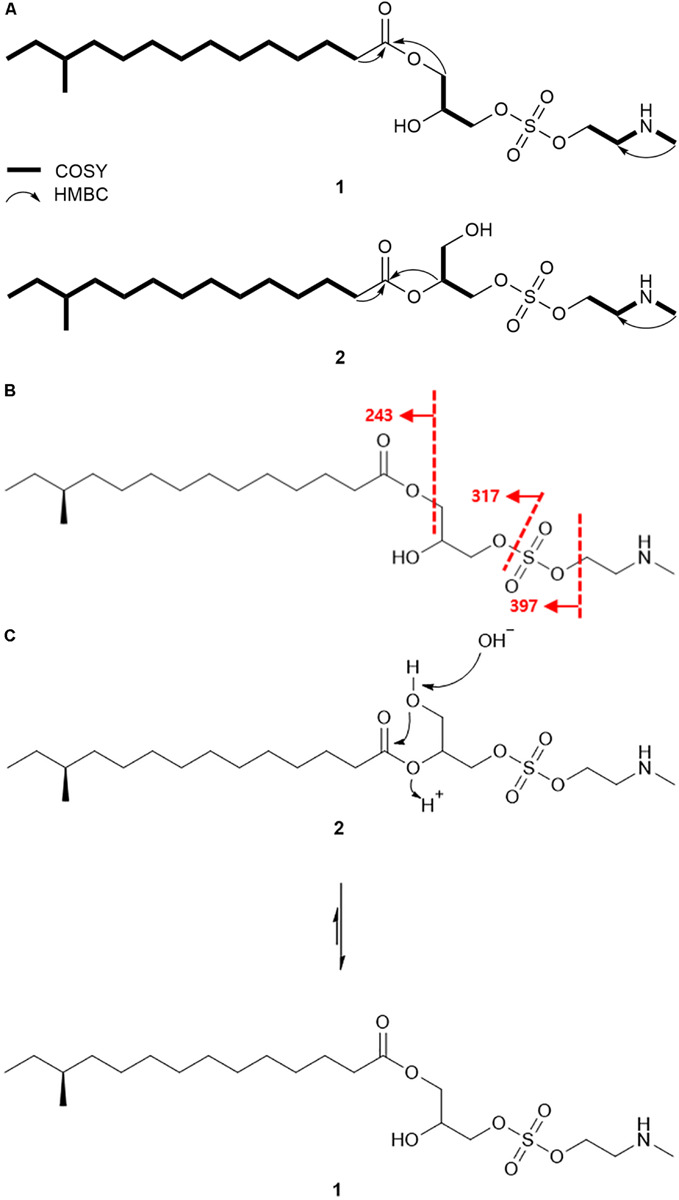
Detailed analysis of planar structures of lenzimycins. **(A)** Key ^1^H-^1^H COSY and HMBC correlations of lenzimycins A and B (**1**-**2**). **(B)** ESI-HR-MS/MS fragmentation of lenzimycin A (**1**). **(C)** Rearrangement between lenzimycins A and B (**1**-**2**).

Based on the COSY correlation analyses, three partial structures were elucidated. An array of COSY correlations from H_2_-2 (δ_H_ 2.30) to the terminal methyl protons H_3_-14 (δ_H_ 0.84) assembled a long lipophilic chain. The branch methyl group 12-Me (δ_C_ 19.2) was connected to C-12 (δ_C_ 34.4) by COSY correlation between H_3_-12-Me (δ_H_ 0.83) and H-12 (δ_H_ 1.28). This connectivity was further confirmed by HMBC correlations from H_3_-12-Me to C-11 (δ_C_ 36.6), C-12, and C-13 (δ_C_ 29.5). The HMBC correlation between H_2_-2/C-1 (δ_C_ 173.8) finally identified this substructure as 12-methyltetradecanoic acid. Consecutive COSY correlations from H_2_-1′ (δ_H_ 4.11) to H_2_-3′ (δ_H_ 4.00 and 3.90) through H-2′ (δ_H_ 3.98) established the three-carbon spin system from C-1′ (δ_C_ 64.5) to C-3′ (δ_C_ 68.1). Based on the ^13^C chemical shifts of C-1′, C-2′ (δ_C_ 69.1), and C-3′, these carbons were deduced to bear oxygen, thus confirming the second partial structure as glycerol. COSY correlation between H_2_-1″ (δ_H_ 4.19) and H_2_-2″ (δ_H_ 3.15) established the connectivity between C-1″ (δ_C_ 61.6) and C-2″ (δ_C_ 49.7). A broad singlet NH proton (δ_H_ 9.64) showed COSY correlations with H_2_-2″ and *N*-methyl protons (δ_H_ 2.70), thereby elucidating *N*-methyl ethanolamine which was supported by HMBC correlation from *N*-methyl protons to C-2″ (Figure 3A).

HMBC correlations further assembled these partial structures. The H_2_-1′/C-1 HMBC correlation secured the connectivity between 12-methyltetradecanoic acid and glycerol through an ester linkage at the C-1′ position, and explained the molecular structure with C_18_H_35_O_4_, which in combination with *N*-methyl ethanolamine (C_3_H_8_NO) accounted for C_21_H_43_NO_5_ of **1**. Furthermore, the IR data showed a strong absorption at 1464 cm^–1^ (Pretsch et al., 2008), thereby indicating the presence of a sulfate group, which was speculated to fit between the oxygenated terminal at C-3′ and C-2″ by placing the sulfur and two oxygen atoms. This addition connected glycerol and *N*-methyl ethanolamine thus completed the planar structure of **1** (Figure 3A).

The NMR-based structure of lenzimycin A (**1**) was confirmed by HR-ESI-MS/MS analysis. C-O at C-3′ and S-O bond connected at C-1″ were fragmented to yield the ion at *m/z* 397 by losing *N*-methyl ethanolamine; subsequently, it identified an ion at *m/z* 317 which represented the loss of the sulfate group along with *N*-methyl ethanolamine. Moreover, the mass difference between *m/z* 397 and 317 also confirmed the identity and location of a sulfate group, whereas, 12-methyltetradecanoic acid was represented by the ion at *m/z* 243 (Figure 3B).

Lenzimycin B (**2**) was obtained as a colorless oil, and its molecular formula was deduced to be C_21_H_43_NO_7_S, [identical to lenzimycin A (**1**)] through HR-FAB-MS data. The ^1^H and ^13^C NMR spectra of **2** displayed high similarity with those of **1** but the chemical shifts of the glycerol substructure (C-1′-C-3′) were noticeably different between these. Lenzimycin B (**2**) showed ^1^H signals at δ_H_ 4.97 (1H), 4.08 (2H), and 3.73 (2H) and ^13^C resonances at δ_C_ 72.5, 63.4, and 59.8 whereas lenzimycin A (**1**) displayed the corresponding protons at δ_H_ 4.11 (2H), 4.00 (1H), 3.98 (1H), and 3.90 (1H) and the carbons at δ_C_ 69.1, 68.1, and 64.5. Analysis of the HSQC NMR spectrum assigned all the C-H one bond correlations. Subsequent analysis of COSY and HMBC correlations assigned the three partial structures, 12-methyltetradecanoic acid, glycerol, and *N*-methyl ethanolamine, identical to **1**. The IR absorption at 1466 cm^–1^ (Pretsch et al., 2008) also indicated the same sulfate group as lenzimycin A (**1**). Although mostly similar HMBC correlations were observed for the entire molecule of **2**, 12-methyltetradecanoic acid was connected to C-2′ through an ester bond based on the crucial H-2′ (δ_H_ 4.97) /C-1 (δ_C_ 173.3) HMBC correlation, which finally determined its structure as a regioisomer of **1** with a different substitution pattern in the glycerol moiety (Figure 3A).

Lenzimycins A and B (**1**-**2**) have two stereogenic centers in their structures. The absolute configuration of C-12 in 12-methyltetradecanoic acid was determined by acid hydrolysis of lenzimycin A (**1**) followed by measurement of the optical rotation. After acid hydrolysis, 12-methyltetradecanoic acid (**3**) was obtained and structurally confirmed by ^1^H NMR and ESI-HR-MS analysis. The optical rotation value ([α]${}_{\text{D}}^{25}$ +5.6) was consistent with that of (*S*)-12-methyltetradecanoic acid ([α]${}_{\text{D}}^{25}$ +5.4) but opposite to the value of (*R*)-12-methyltetradecanoic acid ([α]${}_{\text{D}}^{25}$−5.8) (Hansen et al., 1953), clearly assigning a 12*S* configuration, whereas the other chiral center at C-2′ remained unassigned. During purification and structure elucidation of the lenzimycins, it was observed that lenzimycin B (**2**) was converted to lenzimycin A (**1**) (Figure 3C). This could be due to the rearrangement of the fatty acid chain from the C-2′ to the C-1′ position as reported in other fatty acid glycerides (Biswas and Ganguly, 1960).

Lenzimycins A and B (**1**-**2**) were structurally novel as evidenced by the unprecedented combination of fatty acid, glycerol, sulfate, and *N*-methyl ethanolamine, which has not been reported either as natural products or synthetic compounds. The partial combination of 12-methyltetradecanoic acid and glycerol in **1** reported as aggreceride A (Supplementary Figure S19), showing inhibitory activity against platelet aggregation, has been identified from a *Streptomyces* strain (Ǒmura et al., 1986). However, to the best of our knowledge, fatty acid monoglycerides coupled with a sulfate group have not been reported as natural products, thereby indicating the novelty of the lenzimycins.

### Antibiotic Activity of Lenzimycins

Even though the ecology of the oriental dung beetle *Onthophagus* species, including *O. lenzii*, is not well understood, it is clear that this dung beetle species is exposed to various entomopathogens in their habitat as they utilize microbe-rich feces of herbivores. *B. thuringiensis* is a representative entomopathogenic bacterium that is ubiquitously found in insect ecosystems. From the excretion of *O. lenzii* dung beetle, we isolated a potential symbiont of *Brevibacillus* sp. PTH23 and this bacterial strain seem to produce metabolites that inhibit the growth of entomopathogenic *Bacillus* species. Some metabolic agents isolated from *Brevibacillus* sp. are useful as a broad-spectrum treatment for Gram-positive bacteria, while some are even effective against Gram-negative bacterial infections (Hassi et al., 2012). We investigated the potential antibiotic activity of lenzimycins A and B against the *Bacillus* sp. CCARM9248. The estimated MIC values were 64 μg/mL and 8 μg/mL, respectively. Further, a panel of Gram-positive and Gram-negative bacteria that are mostly well-known nosocomial pathogens, including *E. faecalis*, *E. faecium*, *S. aureus*, *Escherichia coli*, *Pseudomonas aeruginosa*, *Klebsiella oxytoca*, *Klebsiella aerogenes*, *Klebsiella pneumoniae*, *Enterobacter cloacae*, *Salmonella enterica*, and *Bacillus cereus* were also tested to elucidate the broad range applicability of **1** and **2**. The analyses showed markedly narrow-spectrum antibiotic effects as they were effective against only a few *Enterococcus* strains such as vancomycin-resistant *E. faecium* (VREF: *vanA*) with MIC values of 0.5 ∼ 1.0 μg/mL, *E. faecium* ATCC 19434 with MIC values of 8.0 ∼ 16 μg/mL, and an *S. aureus* strain CCARM 0205 with MIC values of 32 ∼ 64 μg/mL (Table 2A).

To further define the anti-enterococcal activity of the lenzimycins, additional MIC tests were performed using a different set of four well-studied clinical isolates of *E. faecalis* that respond differently to the glycopeptide antibiotic vancomycin (Foucault et al., 2010). *E. faecalis* JH2-2 is a clinical isolate which possesses no vancomycin resistance system and is sensitive to glycopeptide antibiotics. *E. faecalis* JH2-2::I is a derivative of JH2-2 containing a transposon, Tn1549, which confers an inducible, VanB-type resistance to glycopeptide antibiotics. The related strain, *E. faecalis* JH2-2::I C1, exhibits a constitutive resistance to glycopeptide antibiotics due to a point mutation in the *vanS* gene of the parent JH2-2::I strain. *E. faecalis* BM4316 is another clinical isolate containing transposon Tn1546 that confers an inducible, VanA-type glycopeptide resistance. VanA- or -B type VRE are distinguished by their susceptibility toward the two glycopeptide antibiotics, vancomycin and teicoplanin. Both VanA- and B-type VREs show high level, inducible resistance to vancomycin, but only the VanA-type is also highly resistant to teicoplanin, while the VanB-type shows teicoplanin sensitivity. This is believed to be due to the differences in how VanS senses the two different glycopeptide structures. VanS in VanA-type strains is activated in the presence of vancomycin, teicoplanin and other structurally unrelated cell wall-targeting antibiotics like moenomycin or bacitracin, while VanS in VanB-type strains is activated only by vancomycin and closely structurally related glycopeptides. The lenzimycins were found to be most active against the vancomycin-sensitive strain JH2-2 but also showed activity against both the inducible VanA- and VanB-type resistant strains, and the constitutively resistant JH2-2::I C1 strain (Table 2B). The growth of the other bacterial strains tested was not significantly affected except those of *B. cereus* CCARM 0002 and *S. enterica* ATCC 14028 which showed a weak inhibition (MIC = 128 μg/mL) (Table 2A).

### Lenzimycins Induce Bacterial Cell Wall Stress

To investigate the activity of lenzimycins against the bacterial cell wall, a disk diffusion assay using a *S. coelicolor* reporter strain was performed. The reporter strain harbors a plasmid pIJ6880 that contains a *sigEp*-*neo* gene fusion construct designed to express the *neo* kanamycin resistance gene from a *sigE* promoter, which is inducible by cell wall stress (Hong et al., 2002). Growth in the presence of kanamycin, therefore, indicates cell wall stress, and this system has previously been used to provide a simple but effective bioassay screening tool to identify compounds which compromise the integrity of the bacterial cell envelope (Truman et al., 2014). In the disk diffusion assay, both lenzimycins A and B (**1**-**2**) stimulated the growth of the reporter strains, thereby indicating the bioactivity via weakening of the bacterial cell envelope (Supplementary Figure S18A). Interestingly, this activity was synergistic with the activity of the peptidoglycan biosynthesis inhibitor vancomycin in *S. coelicolor* which possesses an inducible VanB-type vancomycin resistance gene, *vanJ*, which is natively under the control of a vancomycin inducible promoter (Novotna et al., 2012; Supplementary Figure S18B). The findings suggested a different mode of action for the lenzimycins than that for vancomycin.

## Conclusion

Chemical investigation of a *Brevibacillus* strain associated with the dung beetle, *O. lenzii*, revealed new antibiotic compounds, lenzimycins A and B (**1**-**2**). As the symbiotic interactions in the *O. lenzii* ecosystem have not been studied, it remains unclear whether the lenzimycins are the small molecules bridging microbial interactions in the dung beetle ecosystem. However, based on the antibiotic activity of the lenzimycins against *Bacillus* sp. CCARM 9248, most closely related to the entomopathogen *B. thuringiensis*, these compounds were shown to potentially have a protective role against general entomopathogenic bacteria that threaten the beetle’s health. Structural elucidation of the lenzimycins A and B (**1**-**2**) revealed that these unique antibiotics comprised unprecedented combination of a fatty acid, a glycerol, a sulfate, and an *N*-methyl ethanolamine moiety, which has not been reported in any natural and/or synthetic chemical compounds. Additionally, these new natural products exhibit antibiotic activity against some nosocomial human pathogenic bacteria including certain strains of *Enterococcus* that are resistant to the front-line glycopeptide antibiotic, vancomycin. Collectively, these results suggested the pharmaceutical potential of the lenzimycins. Furthermore, this study implicated that the chemical studies of insect-associated bacteria could be an effective research direction in the search for sources of novel natural antibiotics.

## Data Availability Statement

The original contributions presented in the study are included in the article/Supplementary Material, further inquiries can be directed to the corresponding author.

## Author Contributions

D-CO conceptualized the study. JA, S-HH, and SK cultured and isolated the compound. B-YK analyzed the phylogeny of the bacterial strains. DW collected the dung beetle specimen. S-HH, DW, JA, JS, and D-CO performed spectroscopic analysis. H-JH, ES, K-BO, and JL designed and performed the microbial activity assays. S-IJ identified the beetle specimen. JA, S-IJ, JS, H-JH, and D-CO wrote and edited the text. All authors contributed to the article and approved the submitted version.

## Conflict of Interest

The authors submitted a patent application for this work. B-YK was employed by the company ChunLab, Inc. The remaining authors declare that the research was conducted in the absence of any commercial or financial relationships that could be construed as a potential conflict of interest.

## Footnotes

1 www.ezbiocloud.net/identify
