# Supplementary material for: Lenzimycins A and B, Metabolites With Antibacterial Properties From Brevibacillus sp. Associated With the Dung Beetle Onthophagus lenzii
Source: Front Microbiol. 2020 Oct 30;11:599911. doi: 10.3389/fmicb.2020.599911 (PMC7661691; doi:10.3389/fmicb.2020.599911)
Supplement: Supplementary file 1 [file Data_Sheet_1.PDF]

Supplementary Material

Lenzimycins A and B, Metabolites With Antibacterial Properties From *Brevibacillus* sp. Associated with the Dung Beetle *Onthophagus lenzii*

Joon Soo An[1], Seong-Heon Hong[1], Elisabeth Somers[2], Jayho Lee[3], Byung-Yong Kim[4], Donghee Woo[1], Suk Won Kim[1], Hee-Jeon Hong[2], Shin-Il Jo[5], Jongheon Shin[1], Ki-Bong Oh[3], Dong-Chan Oh[1,*]

[1]Natural Products Research Institute, College of Pharmacy, Seoul National University, Seoul, Republic of Korea

[2]Department of Biological and Medical Sciences, Faculty of Health and Life Sciences, Oxford Brookes University, Oxford, UK

[3]Department of Agricultural Biotechnology, College of Agriculture & Life Sciences, Seoul National University, Seoul, Republic of Korea

[4]ChunLab, Inc., Seoul, Republic of Korea

[5]Animal Welfare Division, Seoul Zoo, Seoul Grand Park, Gyeonggi-do, Republic of Korea

*** Correspondence:**
Dong-Chan Oh
dongchanoh@snu.ac.kr

Table of Contents

S3: **Figure S1.** ^{1}H NMR spectrum of lenzimycin A (**1**) at 850 MHz in $CDCl_3$-d_1.

Figure S2. ^{13}C NMR spectrum of lenzimycin A (**1**) at 212.5 MHz in $CDCl_3$-d_1.

S4: **Figure S3.** COSY NMR spectrum of lenzimycin A (**1**) at 800 MHz in $CDCl_3$-d_1.

Figure S4. HSQC NMR spectrum of lenzimycin A (**1**) at 800 MHz in $CDCl_3$-d_1.

S5: **Figure S5.** HMBC NMR spectrum of lenzimycin A (**1**) at 800 MHz in $CDCl_3$-d_1.

Figure S6. ^{1}H NMR spectrum of lenzimycin B (**2**) at 800 MHz in $CDCl_3$-d_1.

S6: **Figure S7.** ^{1}H NMR spectrum of lenzimycin B (**2**) at 200 MHz in $CDCl_3$-d_1.

Figure S8. COSY NMR spectrum of lenzimycin B (**2**) at 800 MHz in $CDCl_3$-d_1.

S7: **Figure S9.** HSQC NMR spectrum of lenzimycin B (**2**) at 800 MHz in $CDCl_3$-d_1.

Figure S10. HMBC NMR spectrum of lenzimycin B (**2**) at 800 MHz in $CDCl_3$-d_1.

S8: **Figure S11.** ^{1}H NMR spectrum of (*S*)-12-methyltetradecanoic acid (**3**) at 600 MHz in $CDCl_3$-d_1.

S9: **Figure S12.** HR-FAB-MS data for lenzimycin A (**1**).

Figure S13. HR-FAB-MS data for lenzimycin B (**2**).

S10: **Figure S14.** ESI-HR-MS/MS data for lenzimycin A (**1**).

Figure S15. Actinomyces isolation medium with 1 mL of the bacterial suspension. *Brevibacillus* sp. PTH23 strain appeared to inhibit the growth of the surrounding *Bacillus* sp. CCARM 9248.

S11: **Figure S16.** Neighbor-joining phylogenetic tree based on 16S rRNA gene sequences showing the position of strain PTH23 among species of the genus *Brevibacillus*. Numbers at nodes indicate the level of bootstrap support (>50 %) based on 1000 resamplings. Bar, 0.005 changes per nucleotide position.

S12: **Figure S17.** Neighbor-joining phylogenetic tree based on 16S rRNA gene sequences showing the position of strain CCARM 9248 among species of the genus *Bacillus*. Numbers at nodes indicate the level of bootstrap support (>50 %) based on 1000 resamplings. Bar, 0.005 changes per nucleotide position.

S13: **Figure S18.** Lenzimycins A and B can induce bacterial cell envelope stress (A), and act synergistically with the activity of the peptidoglycan biosynthesis inhibitor vancomycin (B).

S14: **Figure S19.** Structure of aggreceride A

Table S1. Recipes of isolation agar media.

Reference

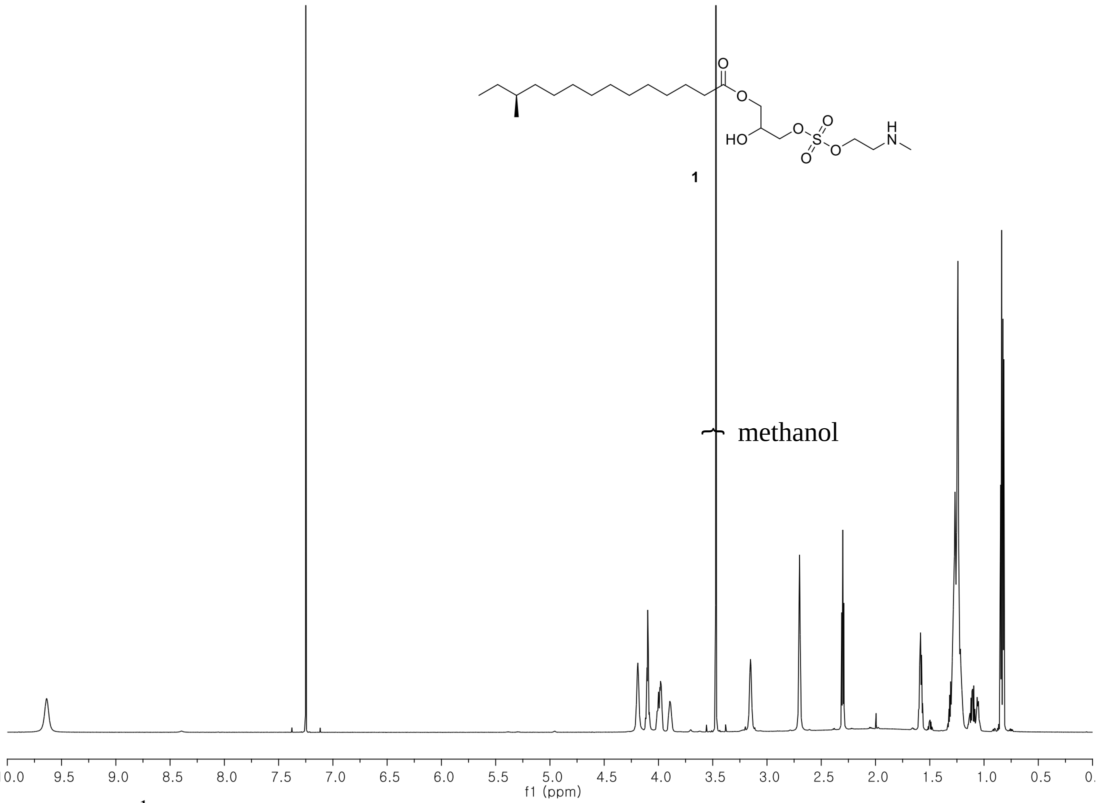

Figure S1. ^{1}H NMR spectrum of lenzimycin A (**1**) at 800 MHz in $CDCl_3$-d_1.

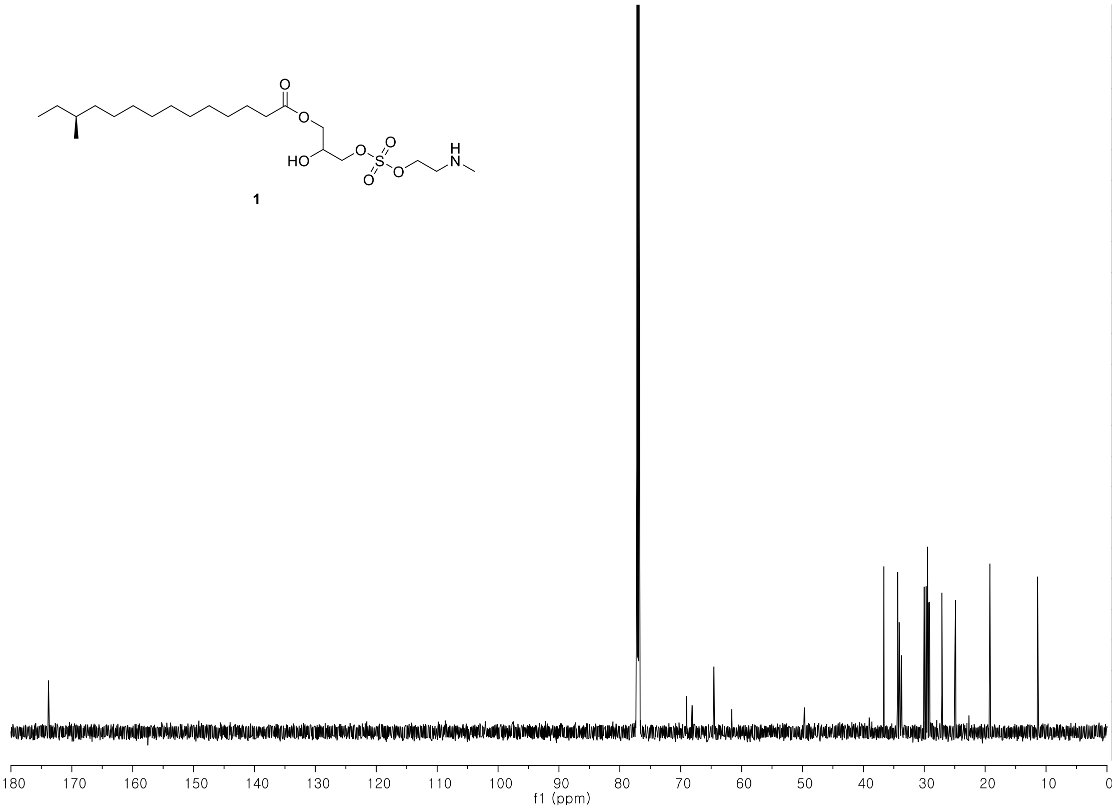

Figure S2. ^{1}H NMR spectrum of lenzimycin A (**1**) at 200 MHz in $CDCl_3$-d_1

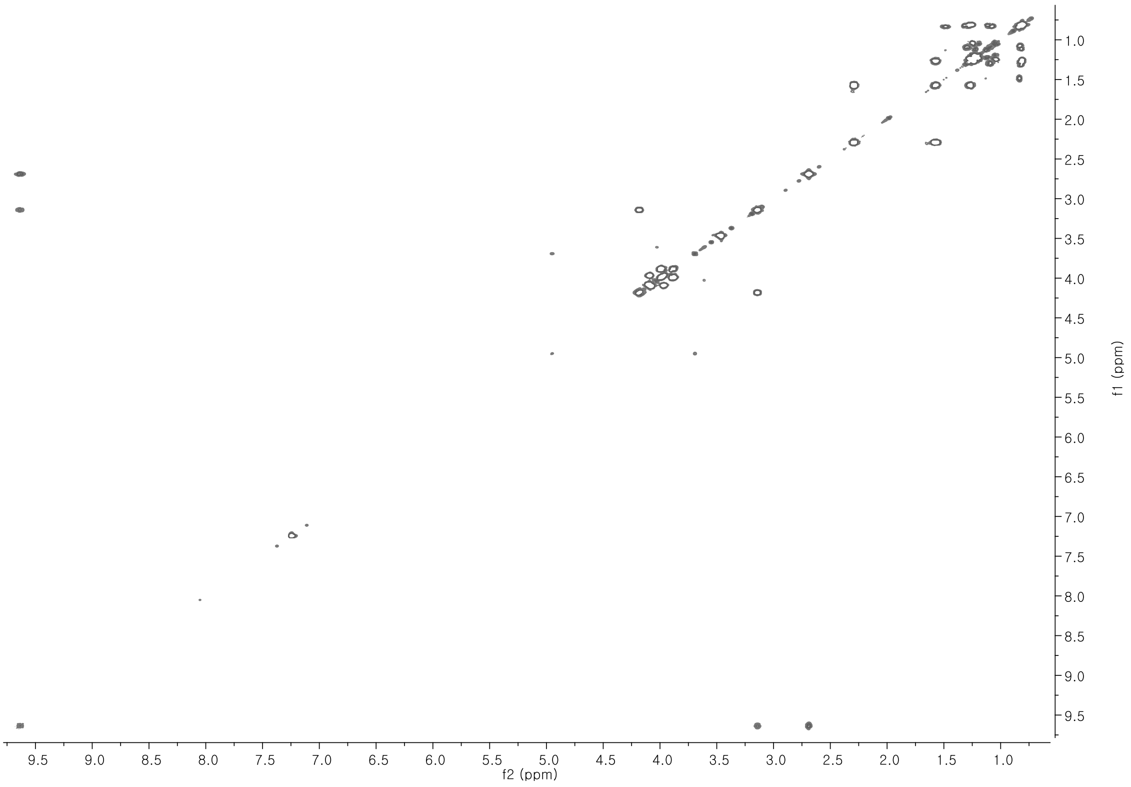

Figure S3. COSY NMR spectrum of lenzimycin A (**1**) at 800 MHz in $CDCl_3$-*d*$_1$.

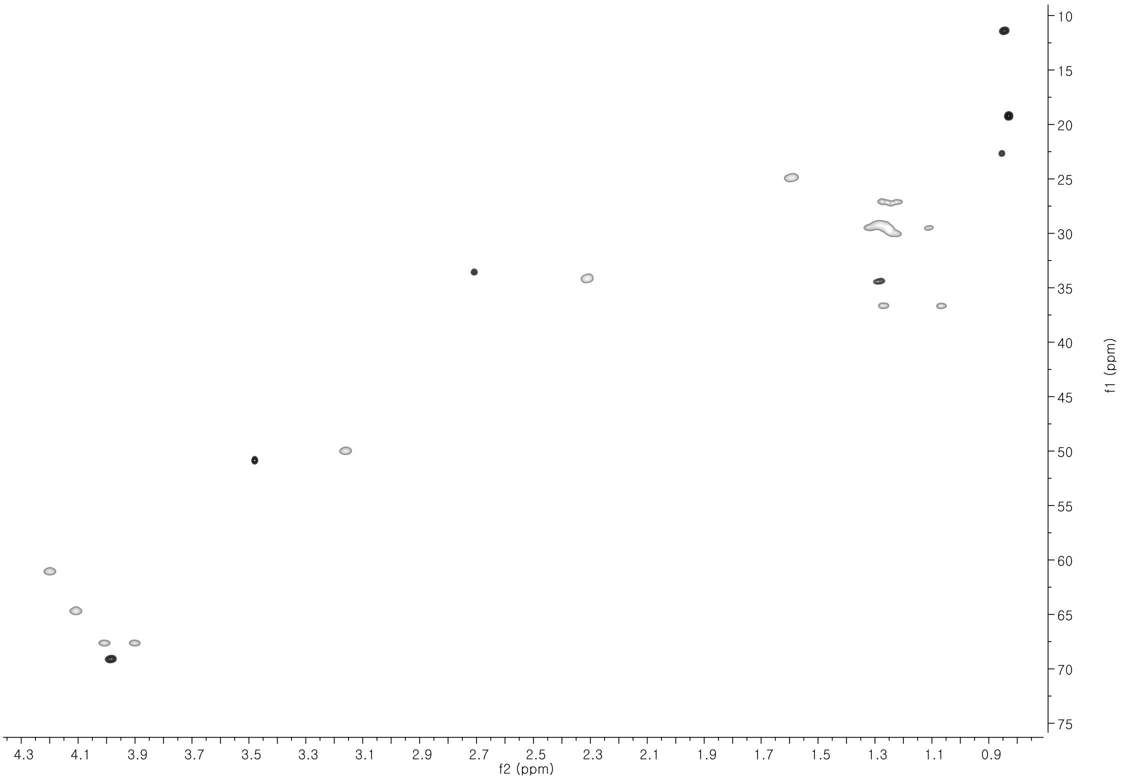

Figure S4. HSQC NMR spectrum of lenzimycin A (**1**) at 800 MHz in $CDCl_3$-*d*$_1$.

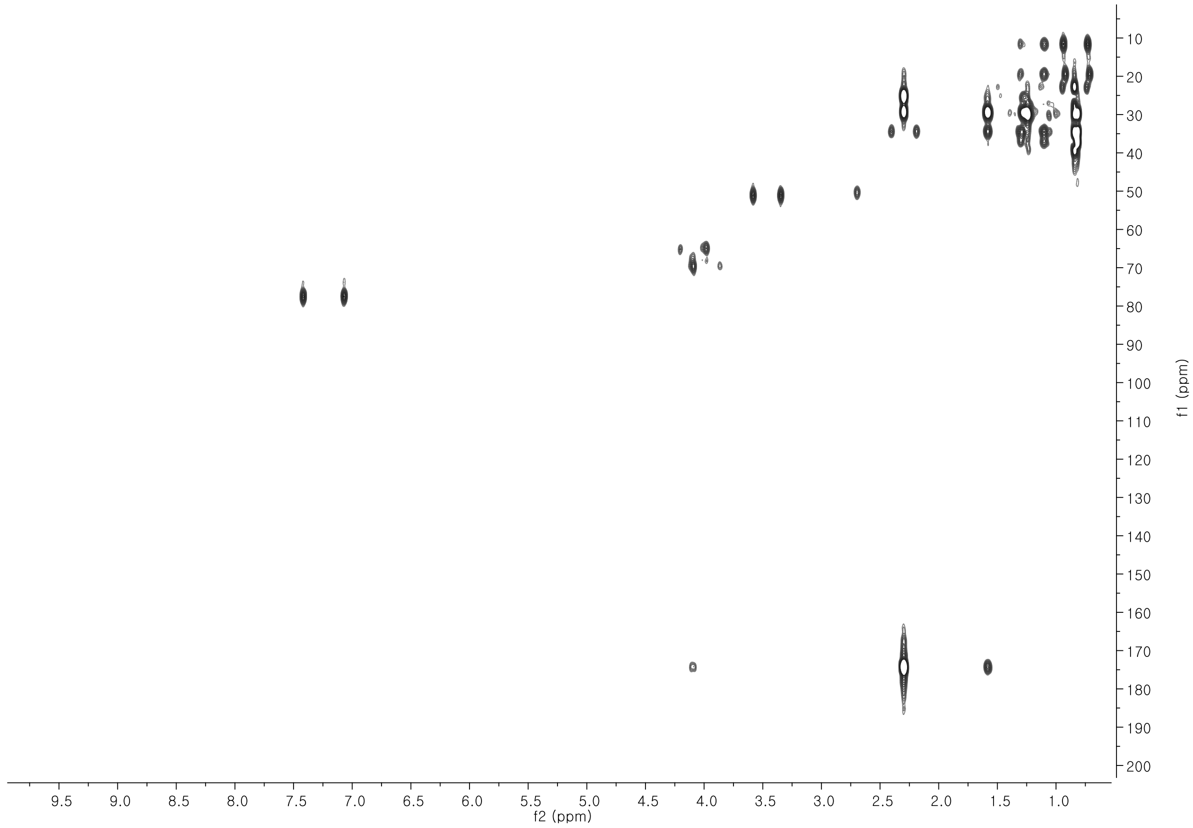

Figure S5. HMBC NMR spectrum of lenzimycin A (**1**) at 800 MHz in $CDCl_3$-d_1.

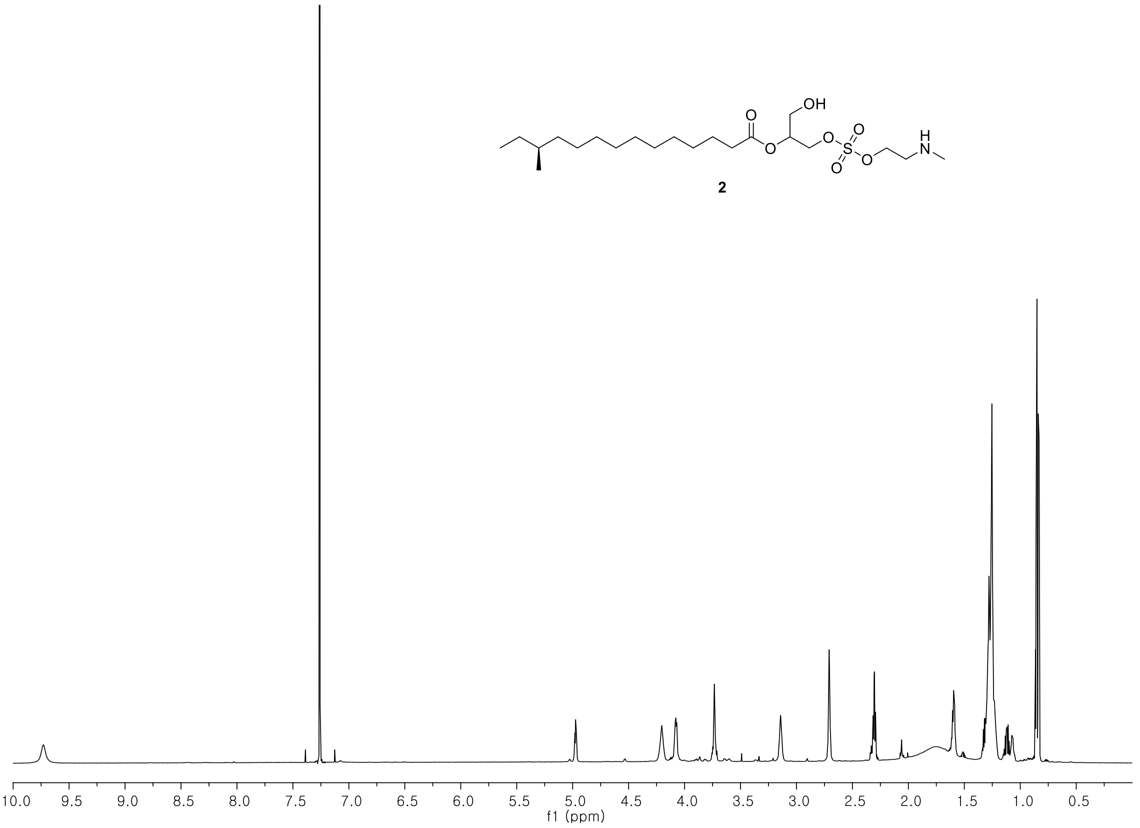

Figure S6. ^{1}H NMR spectrum of lenzimycin B (**2**) at 850 MHz in CDCl3-d_1.

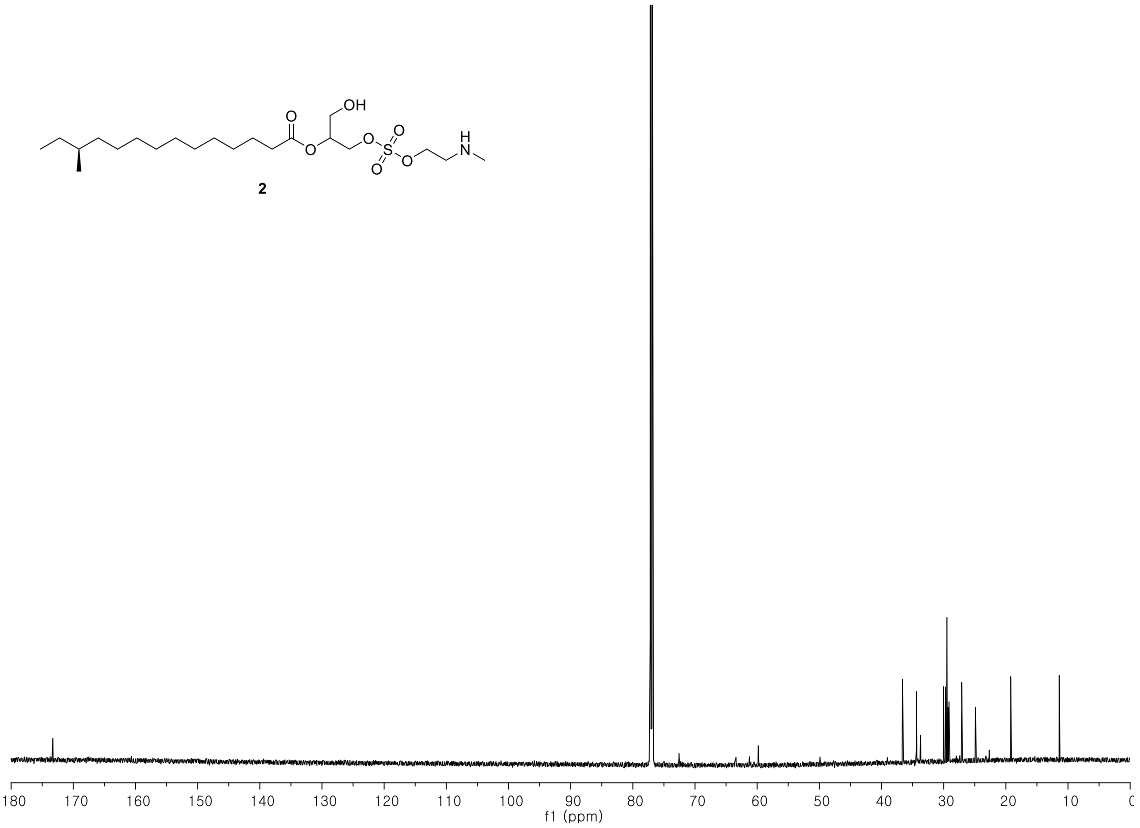

Figure S7. ^{13}C NMR spectrum of lenzimycin B (**2**) at 212.5 MHz in $CDCl_3$-d_1.

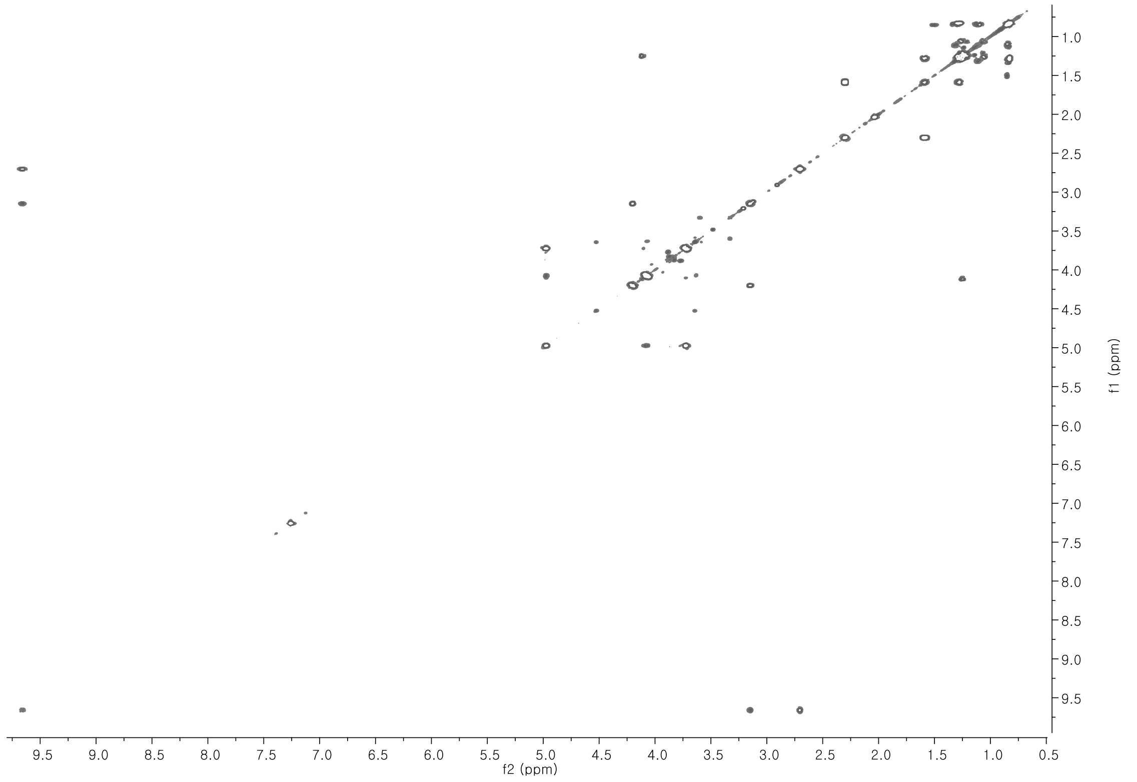

Figure S8. COSY NMR spectrum of lenzimycin B (**2**) at 800 MHz in $CDCl_3$-d_1.

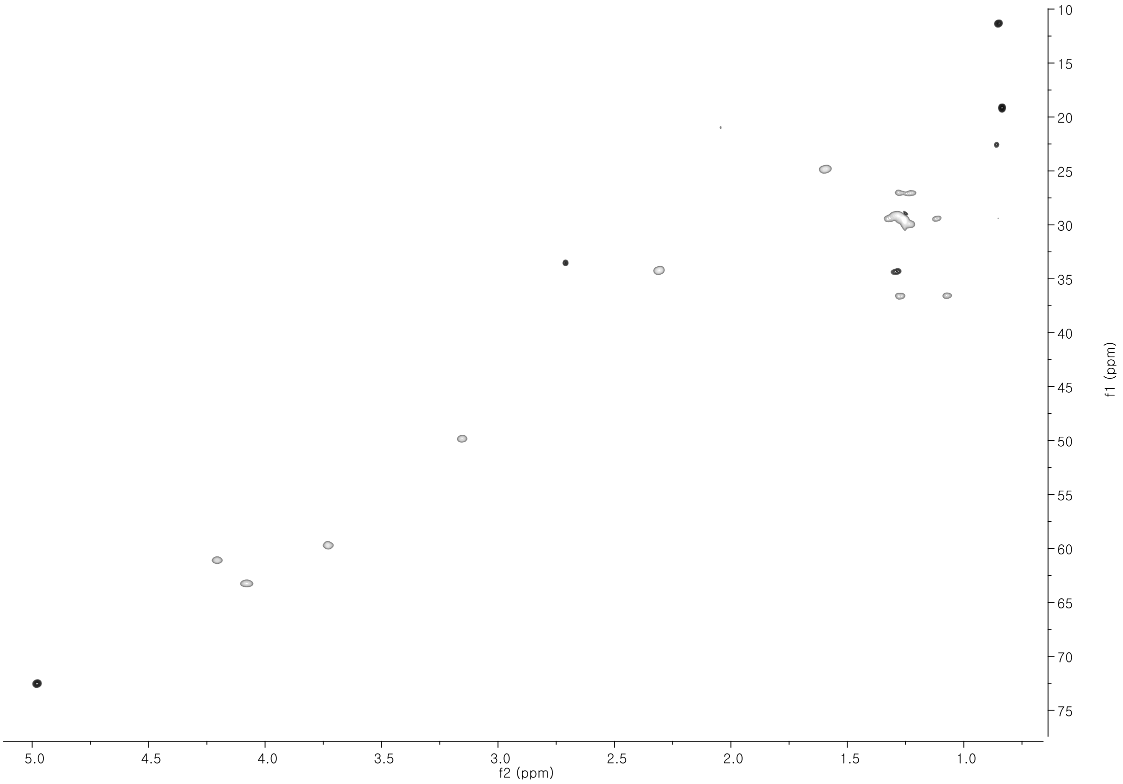

Figure S9. HSQC NMR spectrum of lenzimycin B (**2**) at 800 MHz in $CDCl_3$-*d*$_1$.

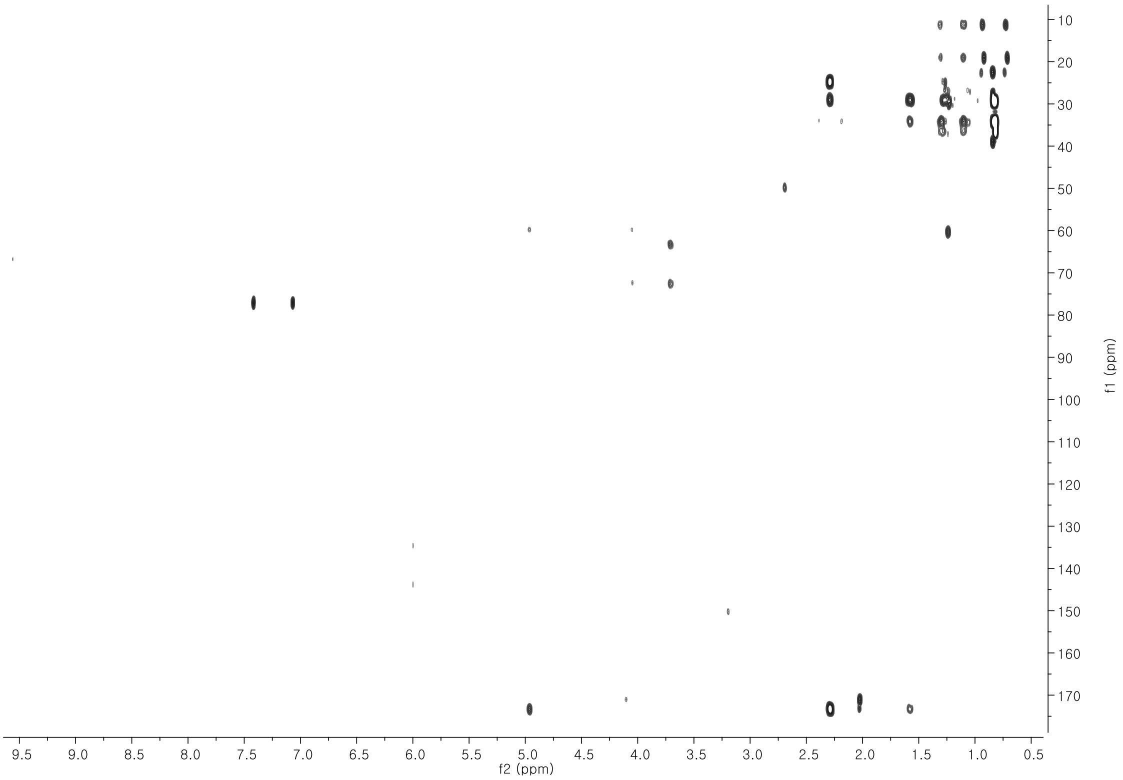

Figure S10. HMBC NMR spectrum of lenzimycin B (**2**) at 800 MHz in $CDCl_3$-*d*$_1$.

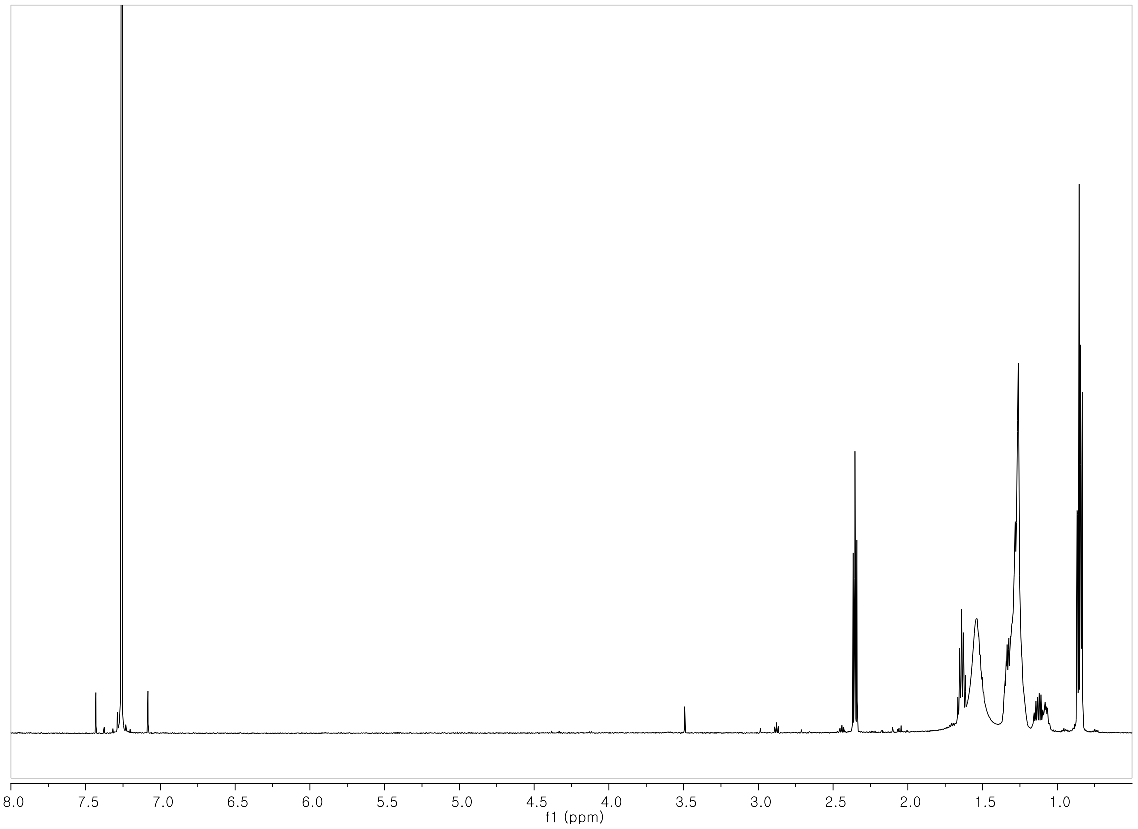

Figure S11. ^{1}H NMR spectrum of (*S*)-12-methyltetradecanoic acid (**3**) at 600 MHz in $CDCl_3$-d_1.

[Elemental Composition]
Data : FAB-Q375 Date : 10-Jan-2018 15:54
Sample: PTH23-453
Note : m-NBA
Inlet : Direct Ion Mode : FAB+
RT : 0.30 min Scan#: (8,21)
Elements : C 100/0, H 100/0, N 5/0, O 10/0, S 5/0
Mass Tolerance : 20ppm, 5mmu if m/z < 250, 10mmu if m/z > 500
Unsaturation (U.S.) : -0.5 - 100.0

Observed m/z	Int%	Err[ppm / mmu]	U.S.	Composition
454.2832	47.6	-5.8 / -2.6	14.5	C 30 H 36 N 3 O
		-8.8 / -4.0	14.0	C 32 H 38 O 2
		+18.9 / +8.6	14.5	C 31 H 36 N O 2
		+3.1 / +1.4	10.5	C 25 H 36 N 5 O 3
		+0.1 / +0.0	10.0	C 27 H 38 N 2 O 4
		-18.7 / -8.5	5.5	C 23 H 40 N 3 O 6
		+9.0 / +4.1	6.0	C 22 H 38 N 4 O 6
		+6.0 / +2.7	5.5	C 24 H 40 N O 7
		-9.9 / -4.5	1.5	C 18 H 40 N 5 O 8
		-12.8 / -5.8	1.0	C 20 H 42 N 2 O 9
		+14.9 / +6.7	1.5	C 19 H 40 N 3 O 9
		+11.9 / +5.4	1.0	C 21 H 42 O 10
		-13.2 / -6.0	10.5	C 27 H 40 N 3 O S
		+14.5 / +6.6	11.0	C 26 H 38 N 4 O S
		-16.2 / -7.3	10.0	C 29 H 42 O 2 S
		+11.5 / +5.2	10.5	C 28 H 40 N O 2 S
		-4.4 / -2.0	6.5	C 22 H 40 N 5 O 3 S
		-7.3 / -3.3	6.0	C 24 H 42 N 2 O 4 S
		+17.4 / +7.9	6.0	C 25 H 42 O 5 S
		+1.5 / +0.7	2.0	C 19 H 42 N 4 O 6 S
		-1.4 / -0.6	1.5	C 21 H 44 N O 7 S

Figure S13. HR-FAB-MS data for lenzimycin A (**1**).

[Elemental Composition]
Data : FAB-R186 Date : 17-Aug-2018 16:37
Sample: PTH23.453-front
Note : m-NBA
Inlet : Direct Ion Mode : FAB+
RT : 0.23 min Scan#: (7,16)
Elements : C 100/0, H 100/0, N 3/0, O 10/0, S 3/0
Mass Tolerance : 20ppm, 5mmu if m/z < 250, 10mmu if m/z > 500
Unsaturation (U.S.) : -0.5 - 50.0

Observed m/z	Int%	Err[ppm / mmu]	U.S.	Composition
454.2833	18.4	-5.7 / -2.6	14.5	C 30 H 36 N 3 O
		-8.6 / -3.9	14.0	C 32 H 38 O 2
		+19.1 / +8.7	14.5	C 31 H 36 N O 2
		+0.2 / +0.1	10.0	C 27 H 38 N 2 O 4
		-18.6 / -8.4	5.5	C 23 H 40 N 3 O 6
		+6.1 / +2.8	5.5	C 24 H 40 N O 7
		-12.7 / -5.8	1.0	C 20 H 42 N 2 O 9
		+15.0 / +6.8	1.5	C 19 H 40 N 3 O 9
		+12.0 / +5.5	1.0	C 21 H 42 O 10
		-13.1 / -5.9	10.5	C 27 H 40 N 3 O S
		-16.0 / -7.3	10.0	C 29 H 42 O 2 S
		+11.6 / +5.3	10.5	C 28 H 40 N O 2 S
		-7.2 / -3.3	6.0	C 24 H 42 N 2 O 4 S
		+17.5 / +8.0	6.0	C 25 H 42 O 5 S
		-1.3 / -0.6	1.5	C 21 H 44 N O 7 S

Figure S12. HR-FAB-MS data for lenzimycin B (**2**).

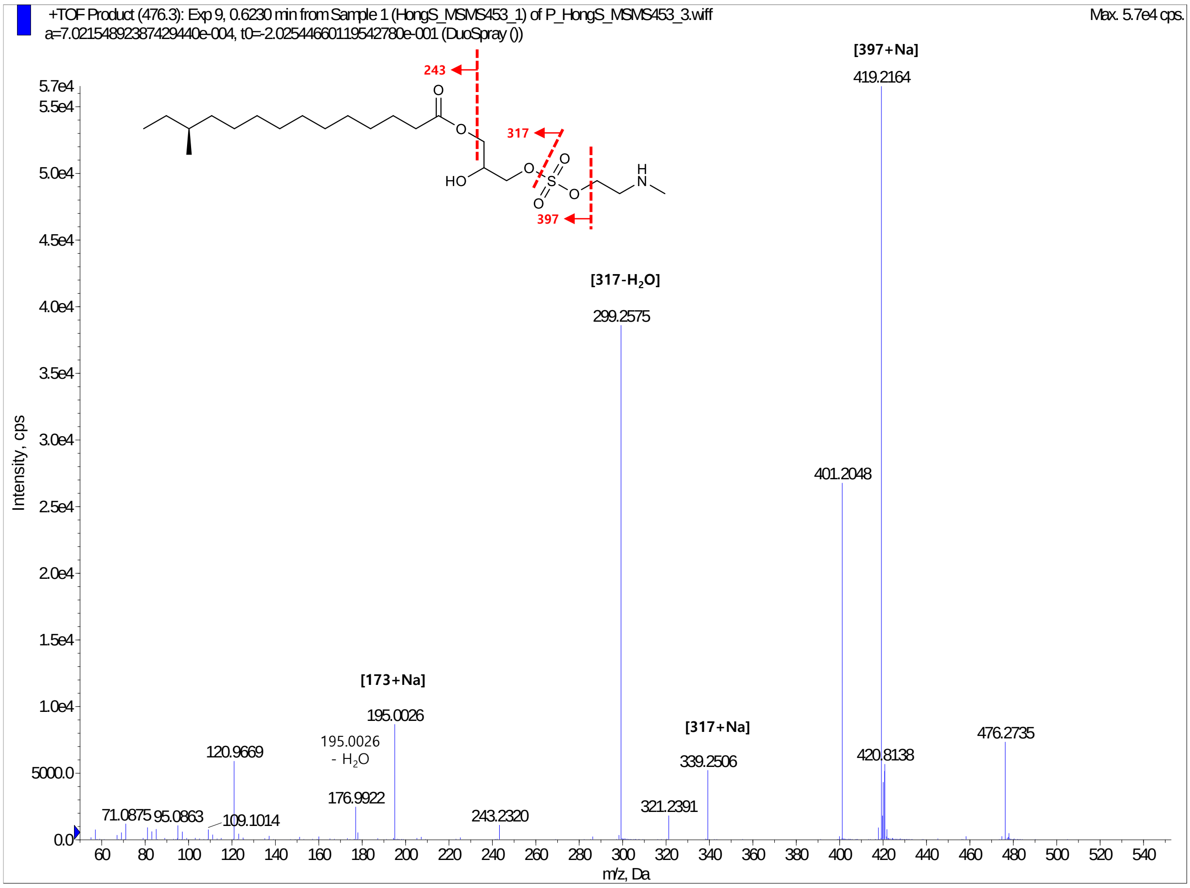

Figure S14. ESI-HR-MS/MS data for lenzimycin A (**1**).

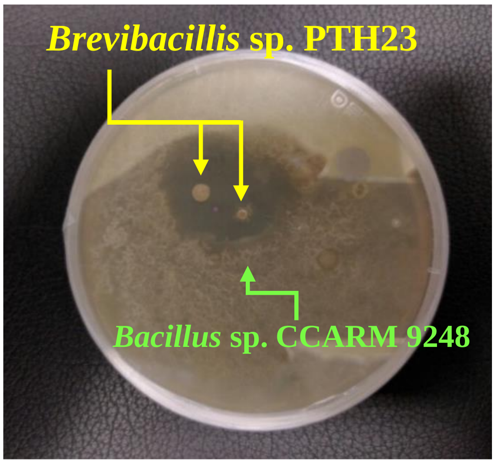

Figure S15. Actinomyces isolation medium with 1 mL of the bacterial suspension. *Brevibacillus* sp. PTH23 strain appeared to inhibit the growth of the surrounding *Bacillus* sp. CCARM 9248.

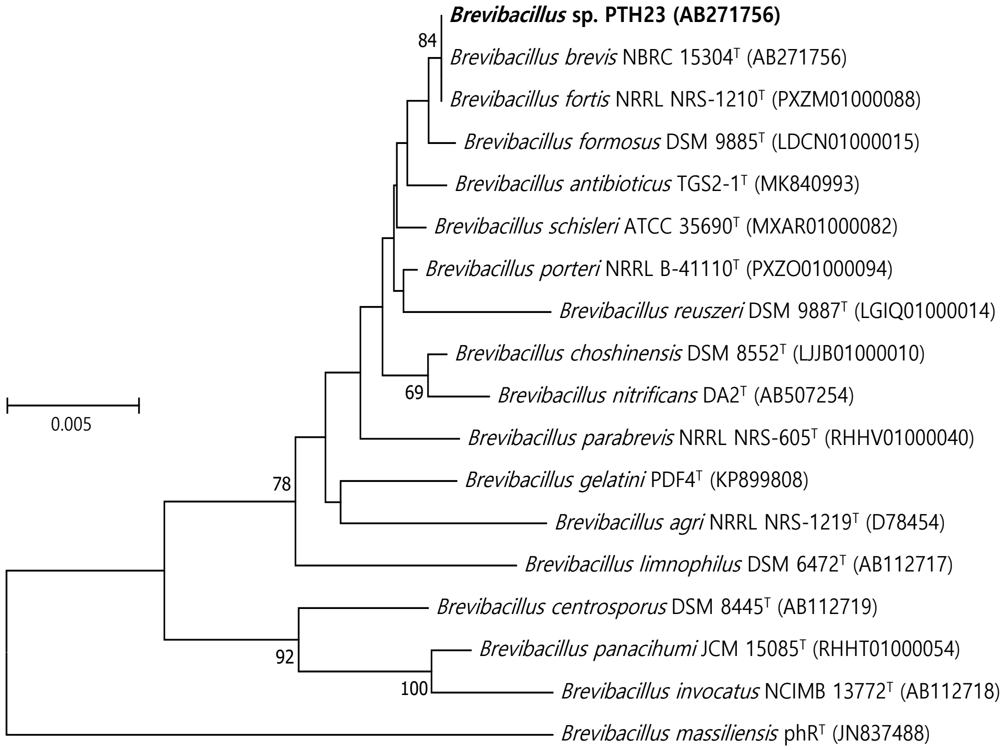

Figure S16. Neighbor-joining phylogenetic tree based on 16S rRNA gene sequences showing the position of strain PTH23 among species of the genus *Brevibacillus*. Numbers at nodes indicate the level of bootstrap support (>50 %) based on 1000 resamplings. Bar, 0.005 changes per nucleotide position.

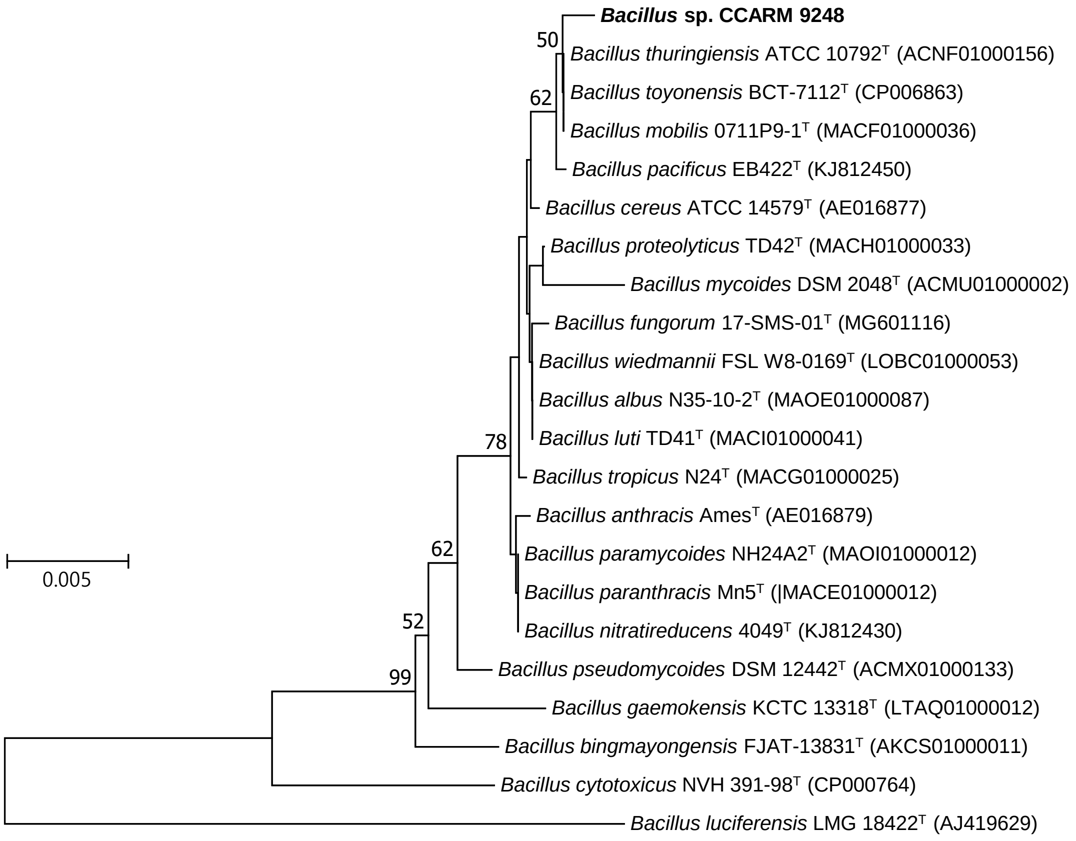

Figure S17. Neighbor-joining phylogenetic tree based on 16S rRNA gene sequences showing the position of strain CCARM 9248 among species of the genus *Bacillus*. Numbers at nodes indicate the level of bootstrap support (>50 %) based on 1000 resamplings. Bar, 0.005 changes per nucleotide position.

(A)

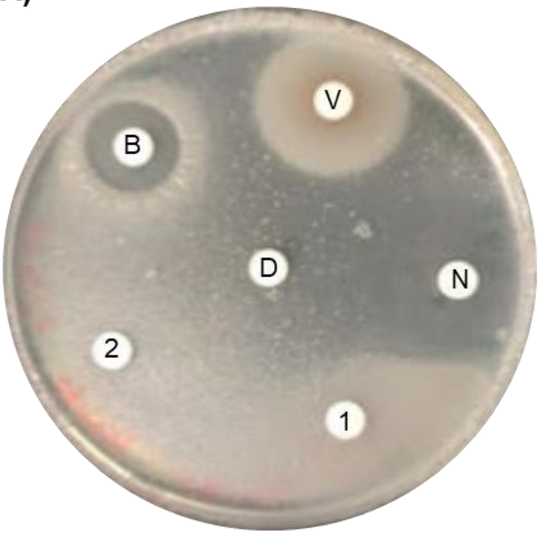

(B)

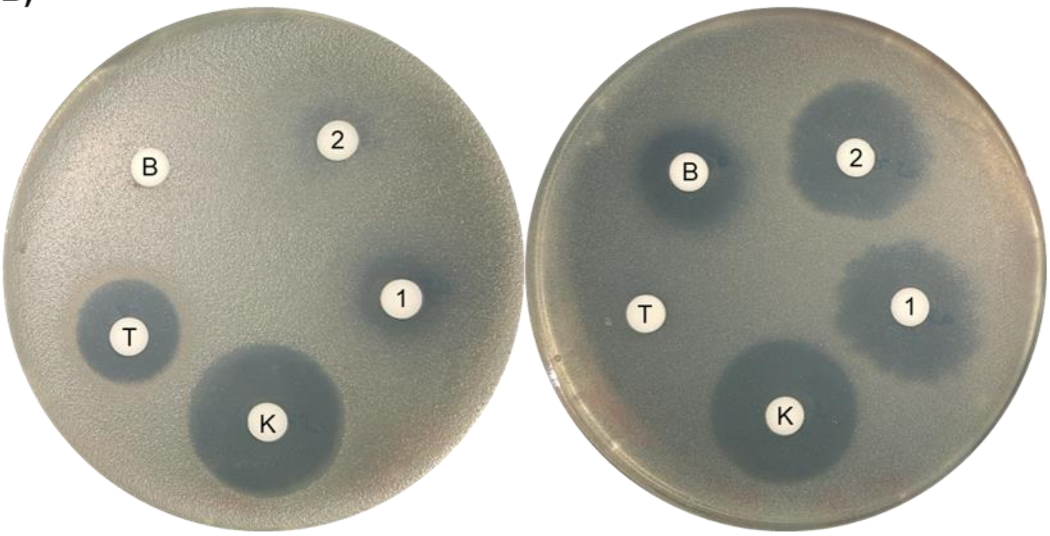

Figure S18. Lenzimycins A and B can induce bacterial cell envelope stress (A), and act synergistically with the activity of the peptidoglycan biosynthesis inhibitor vancomycin (B). In (A), lenzimycin A (1, 50 µg) and lenzimycin B (2, 50 µg) induced kanamycin resistant growth of the sigEp-neo reporter strain around the paper disc. Vancomycin (V, 10 µg) and bacitracin (B, 50 µg) were the positive controls, and novobiocin (N, 50 µg) which inhibits DNA gyrase, was used as a negative control. DMSO (D, 5 µL) was a blank control for activity of the solvent used for the lenzimycins. In (B), the weak inhibition of growth of an indicator lawn of S. coelicolor by lenzimycin A (1, 50 µg) and lenzimycin B (2, 50 µg) is potentiated by the presence of vancomycin in the agar plate (10 µg/ml). Bacitracin (B, 50 µg) was used as a positive control and kanamycin (K, 5 µg) which targets the cell ribosome was used as a negative control. The activity of teicoplanin (T, 5 µg) was antagonized by the presence of vancomycin (Novotna et al., 2012).

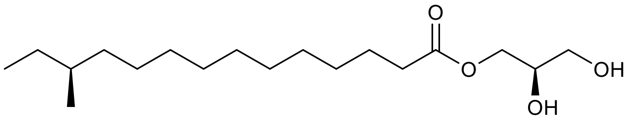

Figure S19. Structure of aggreceride A

Table S1. Recipes of isolation agar media.

	Composition in 1 L of distilled water
ISP1	pancreatic digest of casein 5 g, yeast extract 3 g, agar 18 g
ISP2	yeast 4 g, malt extract 10 g, glucose 4 g, agar 18 g
ISP4	soluble starch 10 g, dipotassium phosphate 1 g<br>magnesium sulfate USP 1 g, sodium chloride 1 g<br>ammonium sulfate 2 g, calcium carbonate 2 g<br>ferrous sulfate 1 mg, manganese chloride 1 mg<br>zinc sulfate 1 mg, agar 20 g
Czapke-Dox	Czapek-Dox broth 35 g, agar 18 g
K	yeast extract 3 g, glucose 2 g, mannitol 2 g, malt extract 5 g,<br>starch 5 g, soytone 5 g, calcium carbonate 1 g, agar 18 g
Chitin-based	chitin 4 g, dipotassium phosphate 3.5 g<br>magnesium sulfate USP 0.75 g, monopotassium phosphate 3.5 g<br>ferrous sulfate 10 mg, manganous chloride 10 mg<br>zinc sulfate 10 mg, agar 18 g
Actinomycete isolation agar	actinomycete isolation agar 22 g, glycerol 5 mL

- All isolation agar media contain cycloheximide 100 mg/L

[Reference]

Novotna, G., Hill, C., Vincent, K., Liu, C., and Hong, H.-J. (2012). A novel membrane protein, VanJ, conferring resistance to teicoplanin. *Antimicrob. Agents Chemother*. 56, 1784-1796.
